# CO_2_ Utilization through Reaction with Alcohols: A Quantum Chemical Study

**DOI:** 10.1021/acs.iecr.5c02744

**Published:** 2025-11-04

**Authors:** Francesca L. Bleken, Klaus J. Jens, Kjell-Arne Solli, Ole Swang

**Affiliations:** † Dept. of Process Technology, 275243 SINTEF Industry, P. O. Box 124, Blindern, Oslo 0314, Norway; ‡ Faculty of Technology, Natural Sciences and Maritime Sciences, 11310 University of South-Eastern Norway, Porsgrunn 3918, Norway

## Abstract

Reactions between alcohols and carbon dioxide have been the subject of numerous experimental studies. While such reactions are roughly thermoneutral, a direct reaction is highly activated. It has been found that certain amines enhance the reaction to the point that it runs its course quickly at or slightly above room temperature. In this contribution, we have used quantum chemical methods to arrive at some suggestions for the mechanism(s) of such reactions. Our results indicate that the addition of either an amine or CO_2_ facilitates the reaction. With CO_2_, the reaction is facilitated to the extent that the calculated activation energy is in accordance with the experimentally reported kinetics. Without such facilitators, all attempts at determining a transition state for the reaction were fruitless.

## Graphical abstract

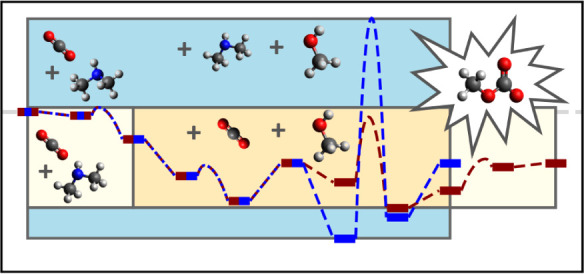

## Introduction

The natural photosynthetic transformation cycle of CO_2_ and water to biological material is essential for the earth’s climate, biodiversity, and human life in terms of food, chemicals, pharmaceuticals, and materials. CO_2_ content in the atmosphere is increasing steadily,^1^ demonstrating an overload of the biological carbon cycle. Hence, there is a need to develop a supplementary technical CO_2_ based carbon cycle in addition to decarbonization (carbon capture) of energy production. The economy thereof could be improved by subsequent utilization of the captured carbon in a Carbon Capture and Utilization (CCU) approach.^2^

CO_2_ is a rather passive molecule and reactions are in general energy unfavorable.^3^ △*G* and △*H* values for small alkyl alcohol carboxylation are, for instance, approx. + 6 and −4 kcal mol^–1^ respectively.^4^ One way of minimizing the reaction energy need is to keep the carbon oxidation number constant at +4 as in the case of organic carbonate synthesis from alcohol and CO_2_^4^. Furthermore, the extraction of the reaction byproduct water using various drying agents promotes carbonate product formation.^5^

Organic carbonates, although today still mainly produced employing harmful phosgene,^6^ attract interest due to being environmental benign products with industrial applications, such as solvents, organic intermediates, agrochemicals, pharmaceuticals, materials, e.g., polyurethanes and polycarbonate,^7,8^ and recently also as advanced battery electrolytes.^9^ Furthermore, the substitution of phosgene as organic carbonate raw material by CO_2_ is of great interest these days.^10^ Carbonic acid esters, a direct derivative of CO_2_, have received less focus as starting materials for the synthesis of organic carbonates.

Carbonic acid monoesters, reported already in 1837 [^11^ and references therein], are unstable^12^ but can be isolated as their respective salts.^13^ Typically, these compounds can be prepared from, e.g., absorption of CO_2_ into aqueous or nonaqueous alcoholic amine or alkanolamine solution at mild conditions.^11,14^ The conversion of carbonic acid ester salts into organic carbonates has so far received little attention^15^ in contrast to their facile synthesis.

Lewis bases react with the polarized carbon of the CO_2_ molecule, which is exemplified in Figure 1. Figure 1a represents a reaction mechanism proposal for monoalkyl ester formation through carbamate alcoholysis in an alcoholic alkanolamine solution.^14^ Here an amine Lewis base connects to CO_2_, forming a carbamate Zwitterion transition state.^16^ In conjunction with a second proton-abstracting amine Brønsted base, a carbamate anion-amine cation pair is formed. Subsequent alcoholysis of the carbamate C–N bond by methyl alcohol results in a carbonic acid ester anion–amine cation pair in addition to free amine.

**Figure 1:**
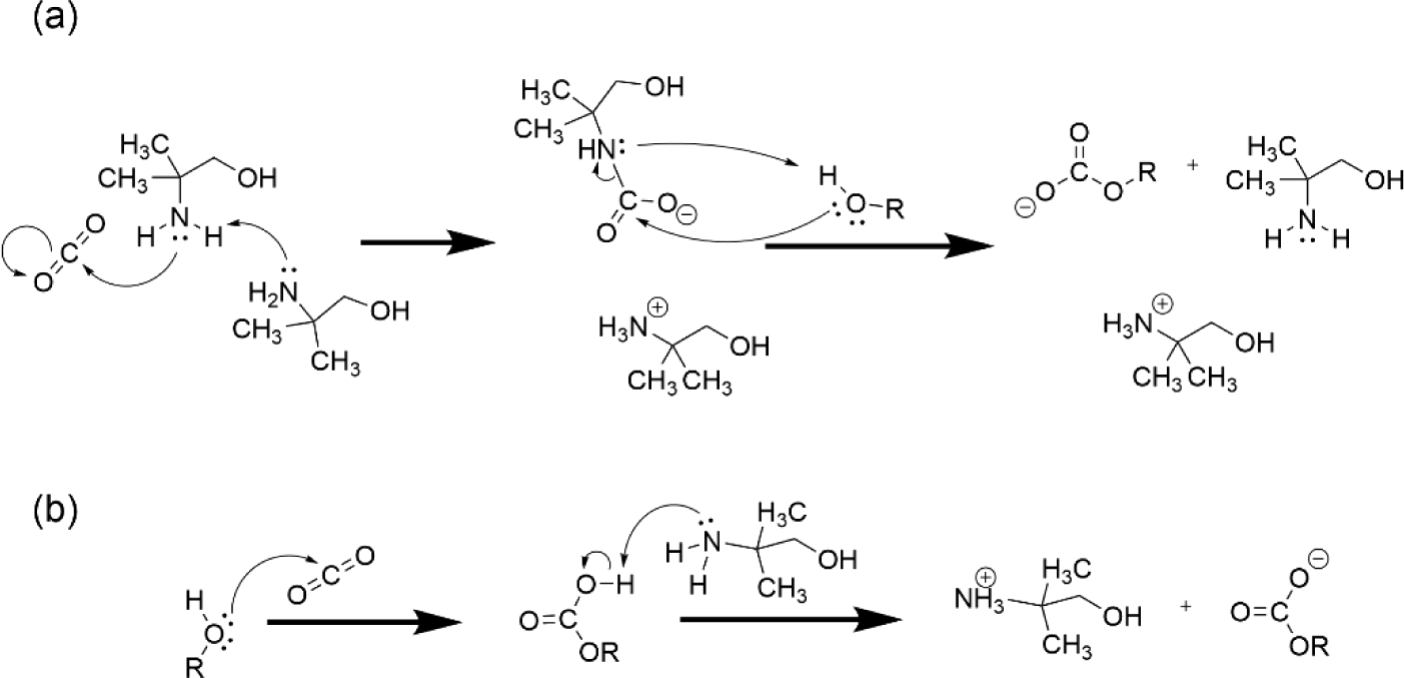
Mechanisms for the reaction between CO_2_ and alcohols analogous to CO_2_ and water/methanol reactions proposed previously in, e.g., references 14 and 17.

Figure 1b shows an alternative reaction scheme for carbonic acid methyl ester synthesis; the reaction of methanol with CO_2_ followed by reaction with alkanolamine.^17^ In this case, the polar methanol reacts with CO_2_ by creating the O–C bond of the methyl carbonic acid monoester, which is stabilized by further reaction with the amine Brønsted base (*vide supra*). Experimentally, these two mechanisms are hard to distinguish.

This contribution provides more insight into the specific reaction mechanism of CO_2_ with alcohol to support the development of the transformation of CO_2_ into organic carbonates via readily available carbonic acid monoester intermediates. We employ quantum chemical modeling of the different mechanisms (*vide supra*) of alkyl carbonate formation to determine which of the possible mechanisms is more probable.

## Computational Details

DFT calculations were carried out with the NWChem^18^ program using the B3LYP functional^19,20^ and aug-cc-pvdz^21^ basis sets. Solvent effects were accounted for using the COSMO^22,23^ approach, with parameters for acetonitrile. The dielectric constant of acetonitrile was chosen to model the solvent since the experimental process proceeds in a highly polarizable but aprotic solvent. In the experimental situation that we address,^24^ the solvent consists of 30% diisopropylamine (δ = 3), 35% methanol (δ = 33), and 35% sulfolane (δ = 43). Eventually, some water (δ = 80) will be present. We have chosen to use parameters for acetonitrile (δ = 37) as a compromise and do not believe that the conclusions presented herein would become different upon changes in epsilon within reasonable limits. In the COSMO calculations, a finer tessellation than the default was employed, as recommended by Marenich et al.^25^ Nudged Elastic Band (NEB)^26^ calculations as well as standard stepwise bond distance variation optimizations were used in initial transition state searches. Final transition state searches were performed with the NWChem optimization modules for saddle points. For all transition states, vibrational frequencies were computed to ensure that the Hessian matrix had exactly 1 negative eigenvalue. Final and initial states for each reaction were optimized from geometries obtained by slightly perturbing the TS in either direction along the corresponding vibrational mode. Two different amine models were used: dimethylamine (DMA) and diisopropylamine (DIPA).

## Results and Discussion

The mechanistic investigations presented herein are inspired by the mechanism for the formation of monomethyl carbonate from CO_2_ and methanol facilitated by diisopropylamine as a solvent, proposed by Wagaarachchige,^24^ and presented as reactions A–C in Figure 2.

**Figure 2:**
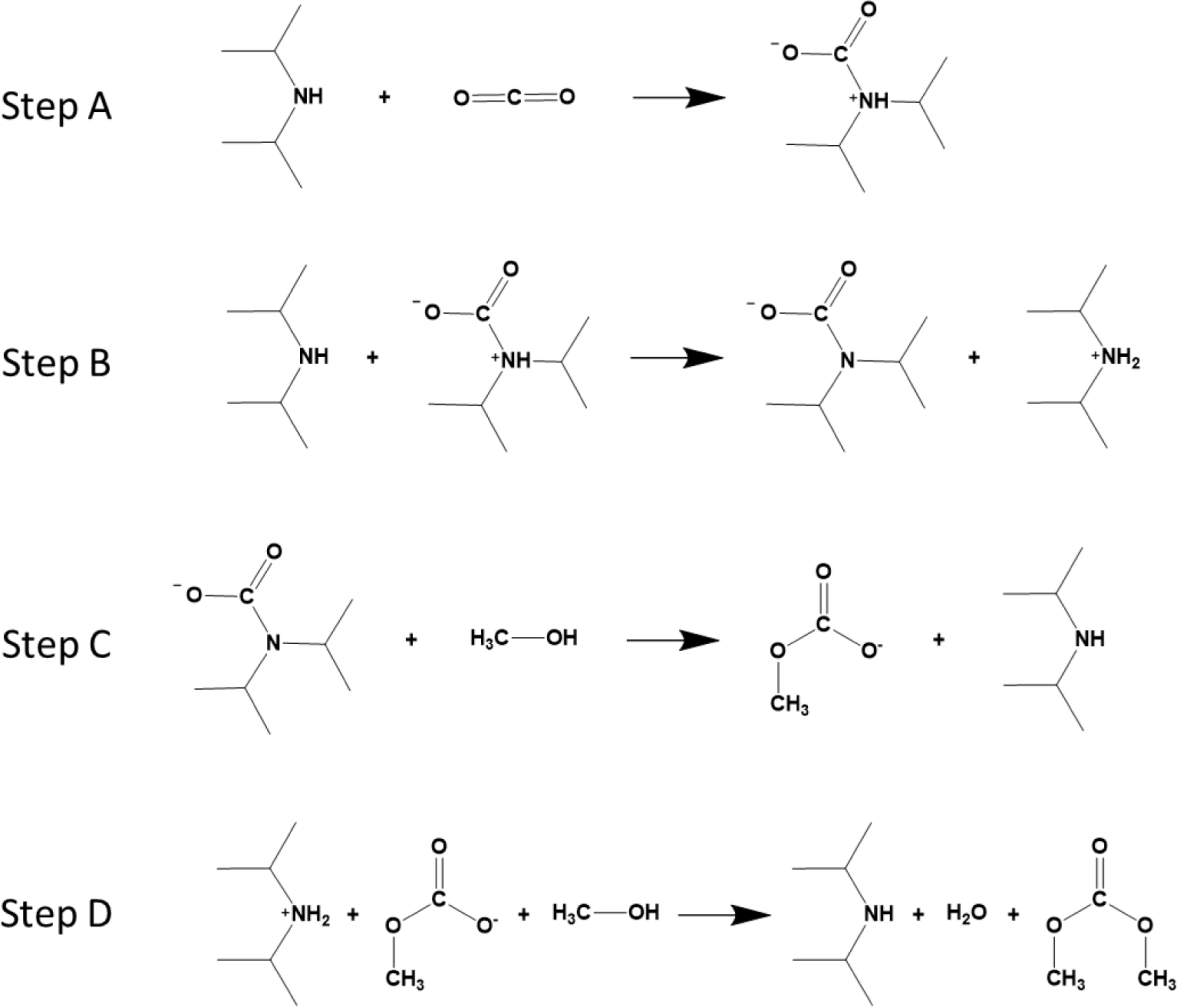
The suggested reaction mechanism from Wagaarachchige et al.^24^ (reactions A, B, and C) and the suggested mechanism for the formation of the dimethyl carbonate (reaction D).

In the following, we report results for each of the reaction steps, with the reservation that no reaction mechanism for Step C could be found without the presence of and interaction with additional species (*vide infra*). The initial, transition, and final states for each reaction are shown as ball-and-stick models in Figure 3. While a 2-dimensional depiction cannot convey all the information of a 3-dimensional reaction, it can be seen that the CO_2_ molecule is bent in all cases where it takes part in the reaction. Overall, it is relevant to note that the reaction proceeds in the same manner irrespective of the amine (choice of the R-group).

**Figure 3:**
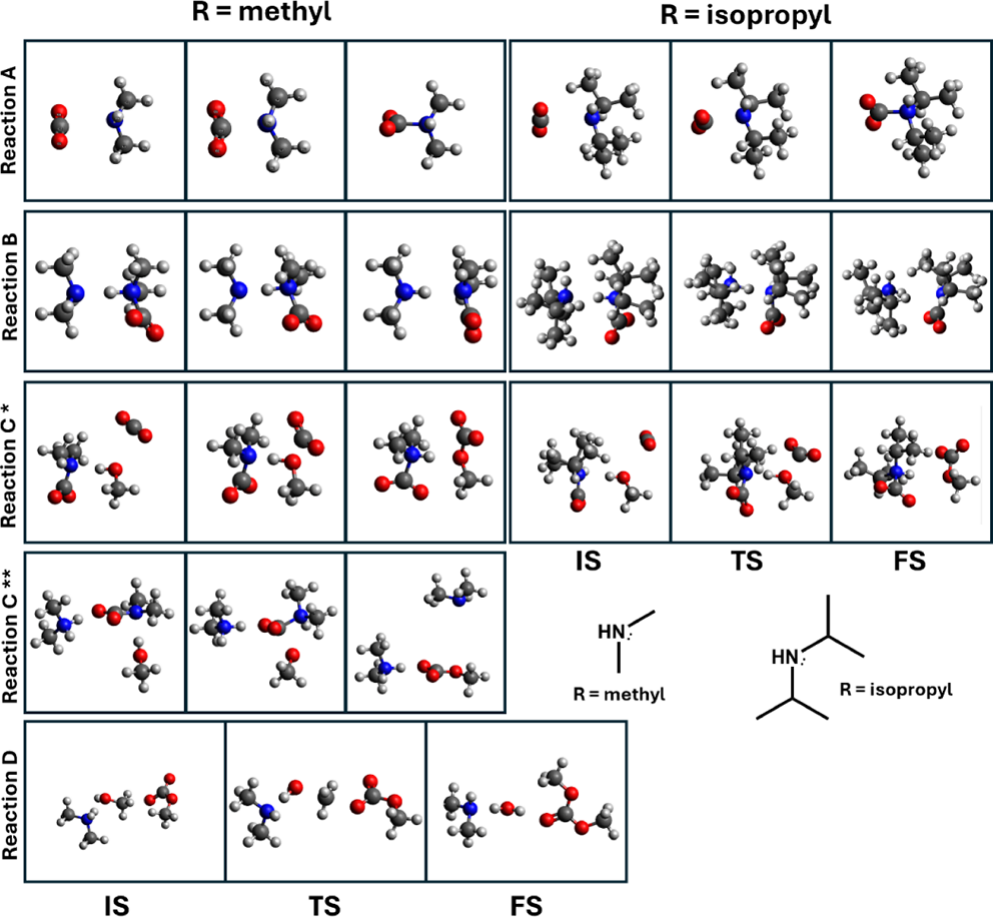
Ball-and-stick figures of initial states (IS), transition states (TS), and final states (FS) of reactions A, B, and C. For reaction C, * indicates the CO_2_-facilitated mechanism and ‡ indicates the amine-facilitated mechanism. The two amine variations are shown at the bottom right.

The reaction energies and activation energies for the activated reactions are given in Table 1 and the full reaction energy profiles are shown in Figure 4.

**Table 1:** Calculated Reaction Energies (Δ*E*)­and Activation Barriers (*E*_a_), in kJ/mol^a^

	DMA (R = CH_3_)		DIPA (R = CH(CH_3_)_2_)	
Reaction	ΔE	Ea	ΔE	Ea
A	–17	6	–10	11
B	–18	7	–28	15
C	–18^b^/15^c^	47^b^/158^c^	–30^b^	66^b^
D	20	147		

a For reaction C, values for the CO_2_- and ammonium-facilitated mechanisms are indicated with * and ‡, respectively. Results for the two amine variations: DIPA = diisopropylamine, and DMA = dimethylamine.

b CO_2_ facilitated.

c Ammonium facilitated.

**Figure 4:**
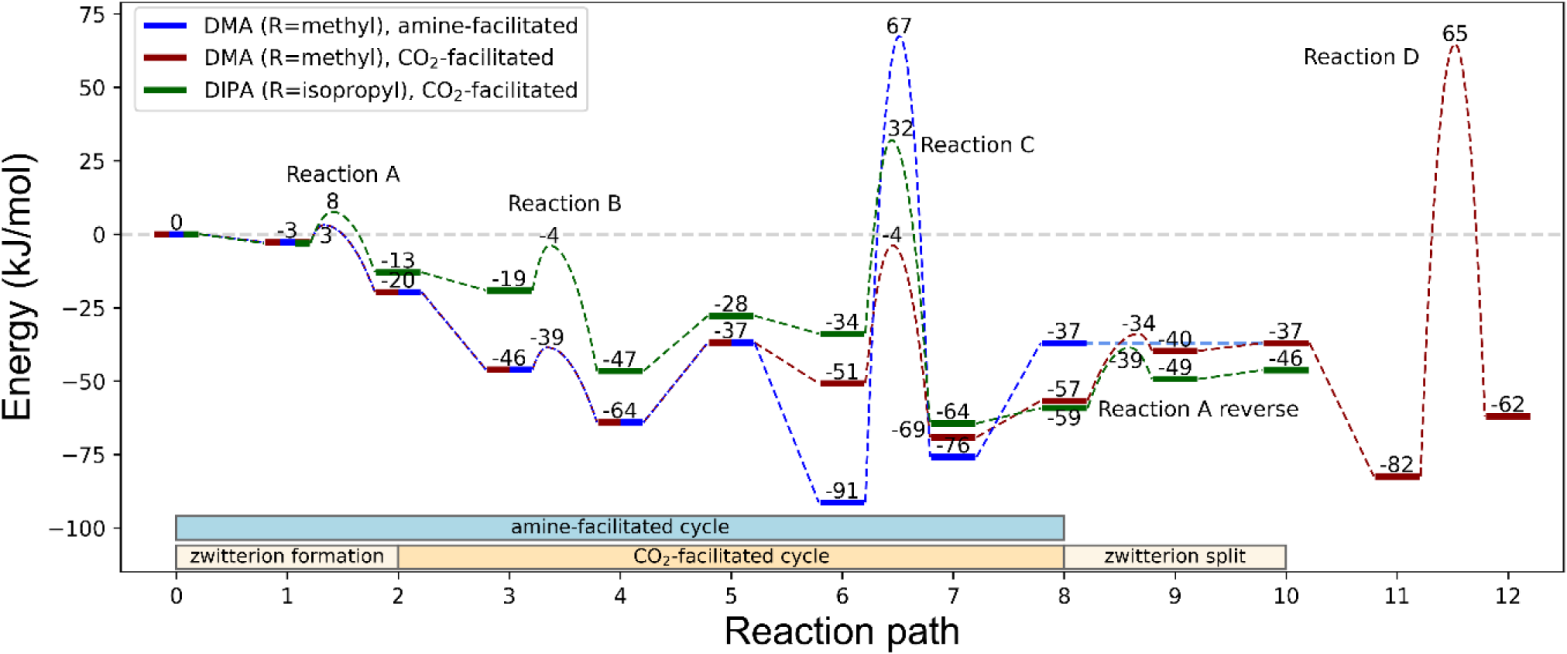
Reaction coordinate diagram for the CO_2_- and amine-facilitated mechanisms. Note that steps 0–1, 2–3, 5–6 represent the formation of the reactant cluster from molecules at infinite separation. Steps 4–5 and 7–8 represent the energy change upon separation of the product cluster to separate molecules. The overall energy of the reaction with DMA (R = methyl) is −37 kJ/mol, and this corresponds to one full cycle of the amine-facilitated cycle (steps from 0 to 8), or one full run of the CO_2_-facilitated mechanism including the “setup-reaction” A and its reverse (steps 0–10). The dashed blue line from step 8 to 10 is to show that the endpoint of the amine-facilitated mechanism at 8 is the same as the endpoint of the CO_2_-facilitated mechanism at 10. The reaction energy of one full cycle of the CO_2_-facilitated mechanism (steps 2 to 8) with DMA is necessarily also −37 kJ/mol. While the overall mechanism facilitated by CO_2_ contains more steps than the amine-facilitated mechanism, the CO_2_-facilitated cycle has fewer elementary steps due to reaction A (setting up the zwitterion catalyst) and its reverse are not part of the cycle. Note that the CO_2_-facilitated and amine-facilitated mechanisms overlap in reactions A and B, i.e., until step 5, at which point the blue and red lines separate.

### The Formation of a Zwitterion (Reaction A)

Looking at Figure 4, it is clear that steps A–D are activated, with the lowest activation energy for step A. The step just prior (0–1) is, in contrast, not activated: The formation of a CO_2_-amine adduct in which the C atom of CO_2_ and the amine nitrogen atom are in close proximity (see Figure 3, reaction A, IS) and ready for reaction A corresponds to a very modest stabilization of 3 kJ/mol, irrespective of the amine. The very small stabilization observed is probably a combination of vdW forces (to the extent that they are described by the hybrid functional) and superposition error. This is in accordance with the reactant molecules still being quite distant (C–N > 3 Å) and the reactant molecules being geometrically very similar to the structures at infinite distance, with the C=O distance in CO_2_ and the C–N–C angle in the amine unchanged. The calculated reaction energies for the formation of the zwitterion are −17 kJ/mol (DMA) and −10 kJ/mol (DIPA) and the corresponding activation barriers are 6 and 11 kJ/mol, respectively. As expected, these barriers are low. Note, however, that the zwitterion formation is only slightly exothermic and the reverse reaction may occur.

### Carbamate Formation (Reaction B)

The starting point of reaction B (see initial states for reaction B at position 3 in Figure 4) is significantly lower in energy with DMA compared to DIPA. This is because creating the adduct that is the starting structure for reaction B, i.e., the zwitterion, is energetically more favorable with the smaller R-group. One might assume a steric effect: but there is no indication of this since the nitrogen–carbon­(CO_2_) distance in the zwitterion is 1.62 Å with DMA and 1.60 Å for DIPA; hardly a significant difference. In the case of DMA, the distance between the two nitrogen atoms (one bearing the proton to be transferred to the other) in the initial state of reaction B is 2.83 Å, while the corresponding value for the DIPA case is 3.03 Å. The products of reaction B are a carbamate and an ammonium ion, and the activation energies are small, 7 kJ/mol (DMA) and 15 kJ/mol (DIPA), as expected for a proton transfer in a solution with a rather high dielectric constant (δ = 37.5).

Danckwerts,^27^ working from kinetic data, proposes that reaction B should be faster than reaction A in the presence of a strong base, hence preventing the reverse of reaction B. In our case, the reaction barriers for reaction B are almost equal in magnitude to those for reaction A, as the base is not so strong. Said et al.^28^ state that “*[t]­he formation of the 1,3-zwitterion (* ... *) is highly unlikely because (* ... *) the associated four-membered mechanism has a high energy barrier [and] (* ... *) is not consistent with the orbital symmetry requirements for chemical reactions*”. They assume that no zwitterion is formed, presumably because they do not find any experimental proof of its existence in the literature. We, however, assume that a zwitterion intermediate may exist transiently, as supported by our computational results, thus circumventing the need for a four-centered mechanism. The need for circumventing a four-centered transition state is also present in Step C, as will be discussed below. The products of reaction B are a carbamate and an ammonium ion. Note that reactions A and B together make up the concerted mechanism proposed by Barzagli et al.^14^ While the presented results indicate that the mechanism is composed of two steps, the possibility that the reaction proceeds in a concerted manner in a liquid phase with proton acceptors available should not be dismissed. The computational work of Said et al.^28,29^ describes a concerted asynchronous reaction mechanism with a hidden zwitterion intermediate in which the extra proton is transferred to the CO_2_-group to form carbamic acid. In the present study, this proton is not transferred to the carbamate oxygen but picked up by a neighboring amine, although it could in principle be picked up by any available base (e.g., from a suitable solvent).

### Carbonate Formation (Reaction C)

Multiple attempts, including numerous NEB calculations, to find a transition state for reaction C as described in Figure 2 were unsuccessful. For such a one-step reaction to proceed, a four-center mechanism is required: A bond is formed between the carbamate carbon and the methanol oxygen, while the C–N carbamate bond is broken and the hydroxyl proton in methanol is transferred to nitrogen, simultaneously. Such a reaction is symmetrically forbidden.^30^ Experiments^24^ show that the reaction does indeed proceed at moderate temperatures, which suggests the involvement of more species in the reaction mechanism. Two routes were investigated, one in which an extra amine functions as a cocatalyst (ammonium facilitated, denoted C^‡^) and one involving an extra CO_2_ molecule (CO_2_ facilitated, denoted C*).

### Ammonium-Facilitated Mechanism (Reaction C^**‡**^)

An extra amine in the form of an ammonium ion can function as a cocatalyst in reaction C, as a facilitator stabilizing the transition state.

The full reaction *cycle* including reactions A, B, and C for this mechanism is shown in Figure 5. The first reaction, reaction A, is the formation of the zwitterion. In reaction B, the zwitterion is deprotonated by an amine. The ammonium ion thus created is involved in the next reaction, as seen in Figure 3, reaction C^‡^. At the starting point, the alcoholic proton of methanol is coordinated to the nitrogen atom that is bound to the molecule, which is subsequently bound to CO_2_. This appears to be a mostly electrostatic interaction, as the corresponding H–N distance is 1.99 Å; further, the methanol O–H bond length of 0.98 Å is not significantly elongated compared to the 0.96 Å found in free methanol. In the transition state, the carbamate nitrogen has already deprotonated methanol. The ammonium ion stabilizes the COO group, so that it is detached from the amine and attached to the methoxy moiety formed by the deprotonation of methanol. In this reaction cycle, the function of the ammonium ion is twofold: It is a byproduct produced in the cycle, and it functions as a cocatalyst during parts of the cycle. The reaction that produces methyl carbonate (reaction C) is endoergic by only 15 kJ/mol but has a quite high reaction barrier of 158 kJ/mol in mechanism C^‡^. This means that the energy of the transition state is 67 kJ/mol higher than the energy of all the reactants prior to the start of the reaction (zero point in Figure 4). This high barrier is not compatible with a reaction that proceeds close to room temperature, and other mechanisms must be investigated.

**Figure 5:**
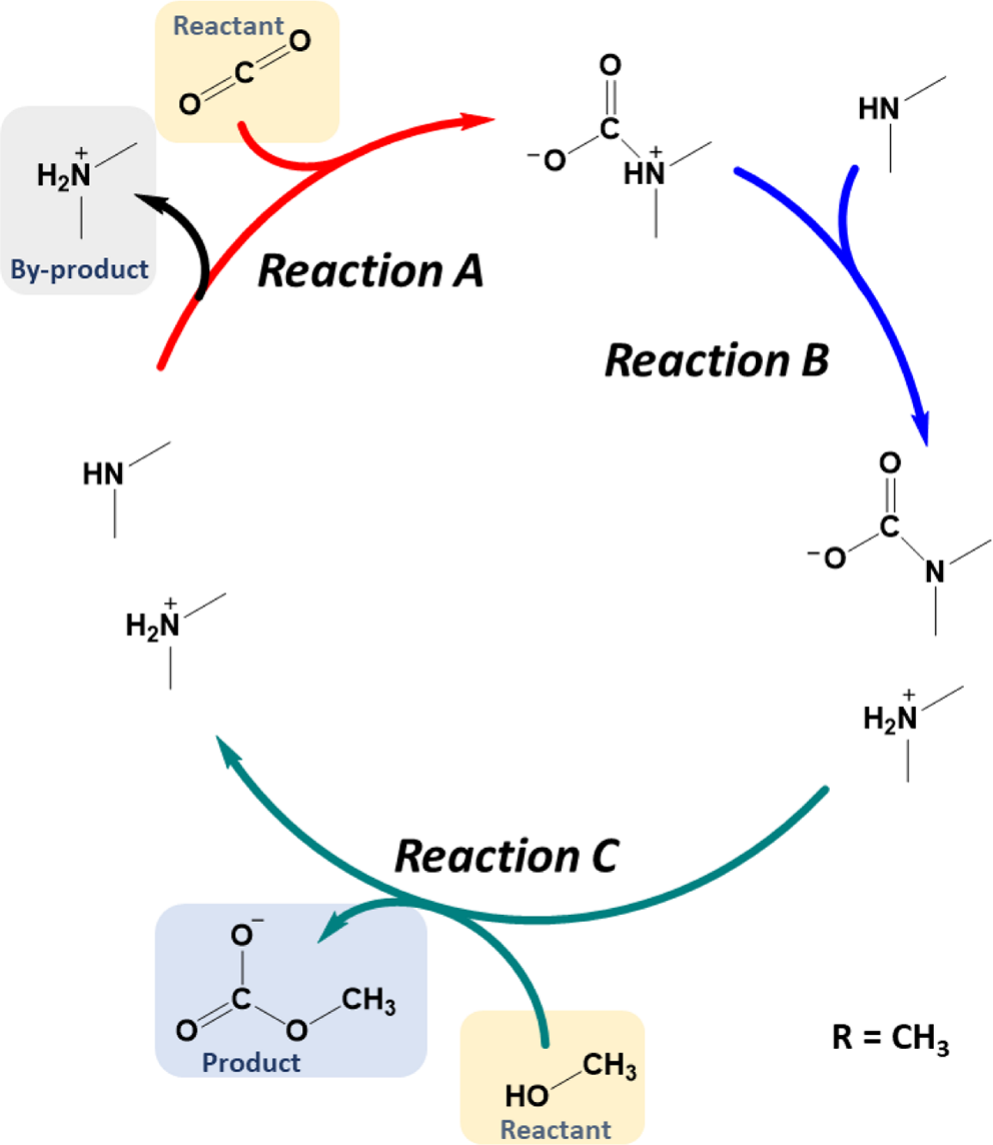
The reaction cycle is facilitated by an ammonium ion, shown with dimethylamine (R = CH_3_).

### CO_2_-Facilitated Mechanism (Reaction C*)

Other species available in the system may also function as facilitators of the reaction. Amine, the molecule used for CO_2_ capture, is available in abundance. Also, CO_2_ itself is present in abundance, limited only by its solubility.

Figures 5 and 6 show that reactions A and B are the same in the two proposed paths, but only reaction B is part of both possible reaction cycles. In the CO_2_-facilitated mechanism, reaction A provides the ammonium carbamate/zwitterion that facilitates further reaction.

**Figure 6:**
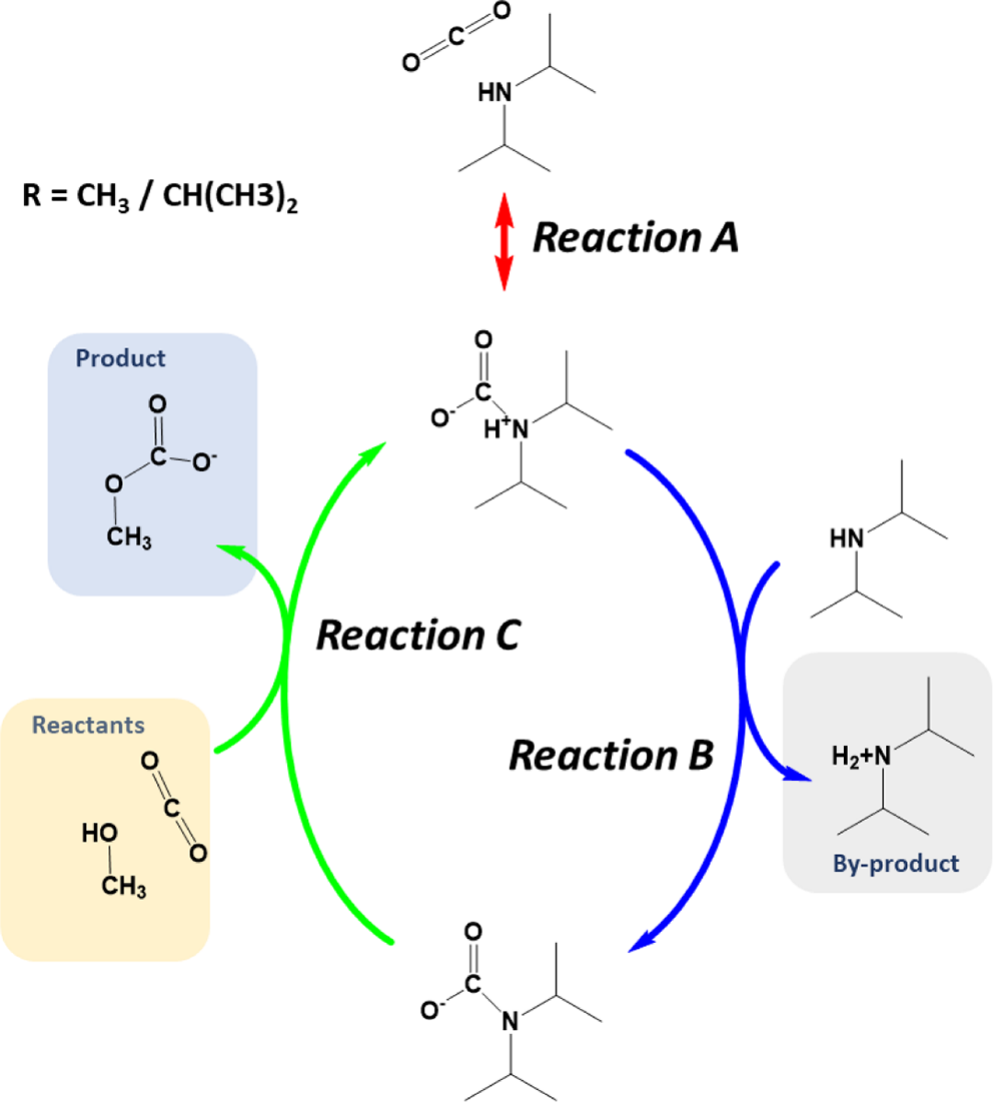
A possible mechanism for monomethyl carbonate formation involving 2 CO_2_ molecules per cycle, one incorporated in carbamate as a facilitator and another as reactant.

In the CO_2_-facilitated reaction path, the carbamate formed in reaction B reacts with both methanol and another CO_2_ in one step to form monomethyl carbonate. In this mechanism, the CO_2_ that has reacted with the amine remains unchanged, and the carbamate acts as a proton acceptor from the hydroxyl group in methanol. Thus, the CO_2_ that reacts with the amine is not the same molecule that ends up in the carbonate product. This can be seen in the transition state shown in Figure 7. The carbamate functions as a proton acceptor, and the methanol O forms a bond to the new CO_2_. The amine has a very different role in the CO_2_-facilitated mechanism compared to the amine-facilitated mechanism. Instead of functioning as a CO_2_ activator, which carries CO_2_ as a reactant, the whole carbamate facilitates the reaction by accepting the extra proton. Energetically, the CO_2_-facilitated mechanism is significantly more favorable than the amine-facilitated mechanism. The Δ*E* of reaction C* is −18 kJ/mol and the activation barrier is 47 kJ/mol with dimethylamine.

**Figure 7:**
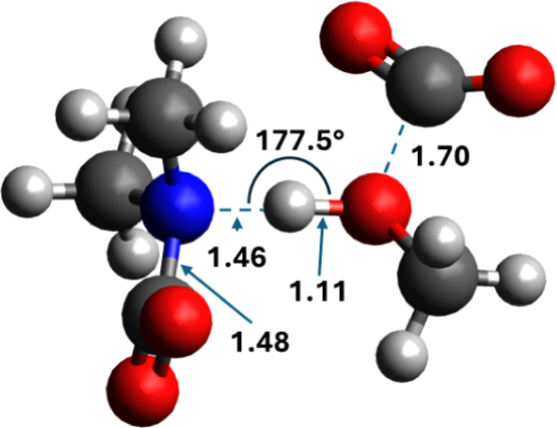
The transition state of the CO_2_-facilitated mechanism for carbonate formation in dimethylamine. Distances in Angstrom.

The CO_2_-facilitated mechanism was also studied with diisopropylamine, resulting in a reaction energy of −30 kJ/mol and an activation energy of 66 kJ/mol. We note that the system with the smaller amine was stabilized the most (Δ*E* = −14 vs −6 kJ/mol) when the 3 molecules were positioned together from an infinite distance and ready for reaction C (position 6 in Figure 4). Again, geometrical considerations indicate that this is more due to electronic rather than steric effects. Indeed, close inspection of the two relevant initial states (IS) in Figure 3 shows that neither CO_2_ nor methanol is in vdW contact with the R-groups on the carbamate. The carbamate N–COO bond length is elongated from 1.40 to 1.43 Å when it is coordinated to methanol and extra CO_2_, irrespective of the alkyl group. Also, the R–N–R angle is almost unchanged, from 122° to 124° for R = diisopropylamine and from 115° to 113° for R = methyl. Table 2 shows relevant bond lengths in the initial state, final state, and transition state for reaction C. Initially, the carbamate N–methanol O distance is larger for DIPA (2.12 Å) compared to DMA (1.93 Å), while the methanol O–H bond length is the same for both variations. The methanol O–CO_2_ distance is only marginally longer when R = isopropyl (3.01 vs 2.97 Å). This suggests that while differences are small, electronic effects make the initial state closer to the final state (and transition state) for DMA, which is in line with a smaller absolute energy of reaction and activation energy. Note, however, that while the actual bond distances in the products are almost identical in the two systems, the end distance between the carbonate and the ammonium proton, the latter originally being the alcohol proton in the reactant methanol, is much shorter for R = methyl (denoted R­(OH)_(MeOH)_ in Table 2).

**Table 2:** Selected Bonding Distances (Å) and Partial Charges (*e*−) as Calculated through Mulliken Population Analysis for Stationary States of Reactions C* and C^‡^ ^a^

Reaction	Species	R(N–H_(MeOH)_)	R(OH)_(MeOH)_	R(O_(MeOH)_–C_(CO2)_)	R(N–C_(CO2)_)	*q*(N)	*q*(O_(MeOH)_)	*q*(C_(CO2)_)
C*R=CH_3_	Reactant	1.93	0.98	2.97	1.43×	–0.67	–0.36	0.38
	TS	1.46	1.11	1.70	1.48×	–0.94	–0.48	0.60
	Product	1.03	1.93	1.41	1.59×	–0.22	–0.55	0.66
C*R=CH(CH_3_)_2_	Reactant	2.12	0.98	3.01	1.43×	0.03	–0.40	0.34
	TS	1.47	1.12	1.77	1.52×	–0.77	–0.59	0.60
	Product	1.02	2.93	1.40	1.60×	0.18	–0.63	0.86
C^‡^R=CH_3_	Reactant	1.99	0.98	3.52	1.41	–0.35	–0.36	0.03
	TS	1.02	2.14	1.98	1.63	–0.37	–0.98	1.15
	Product	1.02	5.61	1.38	5.43	–0.24	–0.56	0.85

a Note that this is the distance between the amine N and the CO_2_ that is not involved in the reaction, i.e., it is the CN bond in the zwitterion that persists.

While one should not rely on their absolute values, differences in partial atomic charges, as calculated by Mulliken population analysis, can be used for a qualitative evaluation of electronic effects. Relevant data are presented in Table 2. The partial charge of the N atom in the reactant configuration is strongly influenced by the size of the R groups of the amine. The negative charge is more localized on the nitrogen with the smaller R groups (−0.67 vs +0.03).

### Comparison of the Two Paths

Figure 4 shows the potential energy diagram for the entire set of reactions. The blue and red lines are for the amine-facilitated and CO_2_-facilitated mechanisms, respectively, with methyl as the R group in the amine. The plot clearly shows that reactions A and B are identical for the two pathways, and the pathways deviate from one another at steps 5–6. In this step the reactant cluster for the next reaction is formed from the species modeled at infinite separation.

It is clear that only the reaction path facilitated with CO_2_ exhibits reaction barriers, in correspondence with reactions proceeding close to room temperature. The barrier for reaction C with DIPA (66 kJ/mol) is 19 kJ/mol higher than with DMA (47 kJ/mol). From a simple Eyring equation argument, these activation energies are in accordance with the experimental observation that the total reaction is fast at a temperature slightly above room temperature. Note that a process based on dimethylamine would face some technical challenges, as it is a gas at STP.

As noted above, a prerequisite for this reaction pathway is the presence of CO_2_. Hence, the solubility of CO_2_ in the relevant solvent at the relevant temperature and pressure may be an important issue when designing a process based on the reaction.

The formation of monomethyl carbonate at 20 °C was demonstrated by Wagaarachchige^24^ for a nonaqueous solvent system containing sulfolane in addition to methanol and amine. The CO_2_ solubility in pure methanol is very low, estimated to be 0.00010 mol/L by extrapolation of data by Lyu.^31^ The sulfolane component, though, contributes to a very high CO_2_ solubility calculated to be 0.75 mol/L at 20 °C based on compiled data from Fogg.^32^ This value can be compared to the CO_2_ solubility in water at 25 °C, 0.035 mol/L.^33^ For a water-based solvent, the presence of amine (20 weight% MEA) does not change the physical solubility of CO_2_ significantly, which is calculated to be 0.031 mol/L based on Henry’s constant given by Zhu^34^ from the correlation by Tsai et al.^35^ It is therefore concluded that the availability of CO_2_ can be fulfilled for the CO_2_-assisted mechanism by the proper choice of solvent.

### Formation of Dimethyl Carbonate (Reaction D)

Methyl carbonate may be reacted further with another methanol molecule to form dimethyl carbonate. The mechanism of this step must be different, as methyl carbonate is a species very different from CO_2_. Searching for possible mechanisms yielded a path based on nucleophilic substitution. Initially, methanol coordinates to dimethylammonium through its hydroxyl hydrogen. Methyl carbonate attacks the methanol methyl group from behind in a classic S_N_2 reaction, while water becomes the leaving group. In accordance with this, the methyl group is close to planar in the transition state. The activation energy is quite high at 147 kJ/mol. Experimental work on ethylene glycol shows that, while the first step is reasonably fast and quantitative at room temperature,^24^ the second step yields only small concentrations of product. Whether that is due to thermodynamics or kinetics is unclear. We would not like to postulate the S_N_2 mechanism on the basis of available data, but rather to propose it as a possibility.

## Concluding Remarks

Experimental studies show that alkyl carbonates can be formed from alcohol and CO_2_ with amine as the solvent. We propose, based on computational studies, a reaction mechanism for the formation of alkyl carbonates from alcohols and CO_2_. Carbon dioxide has a twofold function: (1) as a reactant and (2) as a facilitator for the reaction, by forming a carbamate intermediate that captures the alcoholic proton from the alcohol molecule. Thus, in one reaction cycle, two CO_2_ molecules are involved.

From the point of view of applied CCU, we note that the total reaction has a somewhat lower activation energy with DMA as a solvent/reactant compared to DIPA. Given the important role of CO_2_ as a facilitator in addition to that of a reactant, the solubility of CO_2_ should be addressed by proper choice of solvent composition.

## Data Availability

All the underlying data will be published on zenodo upon publication with doi 10.5281/zenodo.15639178. It includes all calculations and a .ttl file including full data documentation to enable FAIR.
